# Inflammation and Bone Repair: From Particle Disease to Tissue Regeneration

**DOI:** 10.3389/fbioe.2019.00230

**Published:** 2019-09-19

**Authors:** Stuart B. Goodman, Jukka Pajarinen, Zhenyu Yao, Tzuhua Lin

**Affiliations:** ^1^Department of Orthopaedic Surgery, Stanford University School of Medicine, Redwood City, CA, United States; ^2^Department of Bioengineering, Stanford University, Stanford, CA, United States; ^3^Department of Medicine, Clinicum, University of Helsinki and Helsinki University Hospital, Helsinki, Finland; ^4^Orthopaedic Research Laboratories, Stanford University, Stanford, CA, United States

**Keywords:** inflammation, bone healing, bone repair, wear particle disease, osteogenesis

## Abstract

When presented with an adverse stimulus, organisms evoke an immediate, pre-programmed, non-specific innate immune response. The purpose of this reaction is to maintain the organism's biological integrity and function, mitigate or eradicate the injurious source, and re-establish tissue homeostasis. The initial stage of this protective reaction is acute inflammation, which normally reduces or terminates the offending stimulus. As the inflammatory reaction recedes, the stage of tissue repair and regeneration follows. If the above sequence of events is perturbed, reconstitution of normal biological form and function will not be achieved. Dysregulation of these activities may result in incomplete healing, fibrosis, or chronic inflammation. Our laboratory has studied the reaction to wear particles from joint replacements as a paradigm for understanding the biological pathways of acute and chronic inflammation, and potential translational treatments to reconstitute lost bone. As inflammation is the cornerstone for healing in all anatomical locations, the concepts developed have relevance to tissue engineering and regenerative medicine in all organ systems. To accomplish our goal, we developed novel *in vitro* and *in vivo* models (including the murine femoral continuous intramedullary particle infusion model), translational strategies including modulation of macrophage chemotaxis and polarization, and methods to interfere with key transcription factors NFκB and MyD88. We purposefully modified MSCs to facilitate bone healing in inflammatory scenarios: by preconditioning the MSCs, and by genetically modifying MSCs to first sense NFκB activation and then overexpress the anti-inflammatory pro-regenerative cytokine IL-4. These advancements provide significant translational opportunities to enhance healing in bone and other organs.

## Introduction

When exposed to trauma, infection, thermal or chemical injury, or other adverse stimuli, all organisms including humans evoke an immediate, programmed, non-antigen specific immune response to preserve the organism's integrity and re-establish homeostasis (Medzhitov, 2008). This reaction is governed by cells of the innate immune system and defines the acute inflammatory response (Mosser and Edwards, 2008; Chen and Nuñez, 2010). Acute inflammation is the first stage of healing of all tissues, and normally results in repair and regeneration of the damaged structures (Mantovani et al., 2013). If the sequence of events comprising tissue healing is interrupted or dysregulated, the typical healing of host tissue becomes impaired (Gerstenfeld et al., 2003). Furthermore, if the injurious stimulus is not quickly mitigated, either the organism as a whole will succumb (if the injury or resulting response is overwhelming), or the local tissues may progress to a state of chronic inflammation, in which ongoing injury and attempts at repair persist. Thus, the end result of an adverse stimulus may vary from complete restoration of anatomical form and function at one end of the spectrum, to subsequent death at the other extreme; injuries often result in intermediate outcomes including partial tissue regeneration, fibrosis, and/or chronic inflammation.

With regards to bone and soft tissues, the response to injury is no different than for other organs. When bone is subjected to trauma or an adverse stimulus, the resident cells release numerous cytokines, chemokines, and other substances that initiate local vasodilatation and efflux of inflammatory cells from the circulation; a pro-inflammatory cascade of events is launched to terminate the adverse event and initiate the regenerative process (Marsell and Einhorn, 2011; Karnes et al., 2015). Although, numerous cells are directly involved in these ongoing activities, local macrophages, as well as circulating surveillance monocyte/macrophages orchestrate the ensuing series of biological events (Fujiwara and Kobayashi, 2005; Medzhitov, 2008; Nich et al., 2013; Sinder et al., 2015; Kaur et al., 2017). Specialized cellular systems have evolved including pattern recognition receptors (PRRs) to identify chemical motifs from bacteria and infectious agents (so called pathogen-associated molecular patterns or PAMPs) and byproducts of cell death and tissue injury (damage-associated molecular patterns or DAMPS) (Kawai and Akira, 2010). When PRRs are ligated, a system of effector mechanisms including Toll Like Receptors (TLRs), Nucleotide-binding Oligomerization Domain (NOD) and leucine-rich repeat-containing receptors (NLRs), Retinoic acid inducible gene (RIG) receptors, and others transmit these signals through intermediate molecules to upregulate the formation and release of pro-inflammatory substances (Akira and Takeda, 2004; Medzhitov, 2008). These include cytokines e.g., tumor necrosis factor alpha (TNFα), Interleukin 1 beta (IL-1β), IL-6, IL-8, and others, chemokines including macrophage chemotactic protein 1 (MCP-1), macrophage inhibitory protein 1 (MIP-1), reactive oxygen intermediates (such as inducible nitric oxide synthetase or iNOS), and growth factors (such as vascular endothelial growth factor or VEGF, transforming growth factor beta or TGFβ etc.). These cells and substances eradicate invading microbes, limit the injurious stimulus, and recruit more cells to participate in the biological confrontation, and begin the resolution and reparative phases (Medzhitov, 2008; Mosser and Edwards, 2008; Mantovani et al., 2013).

This paper will summarize the important biological processes of inflammation as they relate to bone healing and emphasize the critical intercellular communications that participate in repair of bone subjected to adverse stimuli. *In vitro* and *in vivo* research performed in our laboratory and by others that facilitates bone repair in inflammatory conditions will be highlighted.

## The Biological Reaction to Wear Particles From Joint Replacements: A Paradigm for Acute and Chronic Inflammation

**Figure 1:**
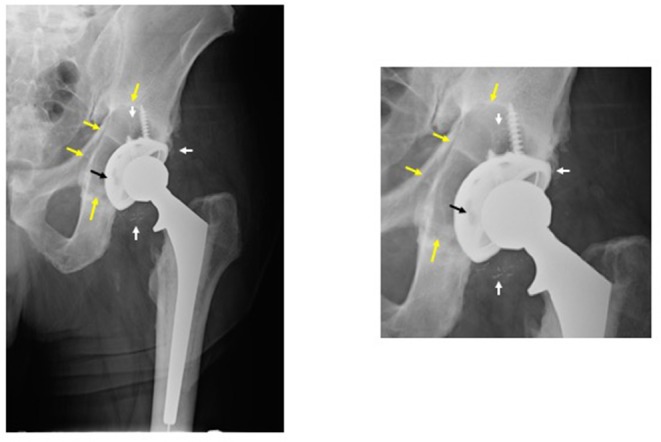
Periprosthetic osteolysis post total hip replacement (THR). The left radiograph shows a hybrid THR with a cemented stem and a cementless cup with screws. The components are well fixed, however, there is polyethylene wear and the metallic cup has fractured adjacent to the screw holes (note: two of the screw holes are larger than they should be and confluent instead of separate—black arrow). The small white radio-dense particles represent metallic debris from the cup (white arrows). There is a large radiolucent black area of bone destruction (osteolysis) (yellow arrows) surrounding the acetabular component. The radiograph on the right is a magnified view of the acetabular area.

Traditionally, most joint replacements have used a bearing couple composed of ultra-high molecular weight polyethylene (UHMWPE), and a metallic or ceramic counter surface. This bearing couple has recently been improved, with the development of enhanced crosslinking of the polyethylene and embedded anti-oxidants, and by reducing the surface asperities and polishing of the countersurface. However, for the first 40 years of joint replacement surgery, the biological reaction to wear particles, and the resultant sterile inflammation and bone loss (known as periprosthetic osteolysis) were the predominant reasons for revision (redo) surgery (Jacobs et al., 2001; Purdue et al., 2007; Gallo et al., 2013) (Figure 1). This subject has been studied extensively by our group and others; numerous *in vitro* and *in vivo* models have been developed to simulate the events of wear particle-induced inflammation.

In general, wear particles stimulate a non-specific macrophage dominated inflammatory reaction characteristic of the innate immune system, in a background fibrovascular stroma (Goodman et al., 1998; Goodman, 2007). The characteristics of the wear particles are important to this reaction: smaller (0.3 to <5–10 μm) irregularly shaped particles of polymers appear to be more inciting of an inflammatory response, compared to ceramic or metallic particles, however this point is controversial (Goodman, 1994; Kaufman et al., 2008; Goodman et al., 2009). Particles ~1 μm or less are the most prominent and reactive ones (Campbell et al., 1995). In addition, to the above particle characteristics, the surface area, surface energy and overall number and volume of particles are key factors in the resultant histological reaction (Shanbhag et al., 1994; González et al., 1996; Green et al., 1998, 2000). Certain metallic particles and byproducts can stimulate both the innate and adaptive immune systems, the latter occurring when the metallic moiety and attached protein function as a hapten (Haynes et al., 1993; Hallab et al., 2001; Caicedo et al., 2008). Indeed, all particles are bound to serum proteins such as albumin, alpha-1-antitrypsin, apolipoprotein, and others, and activate specific cell surface receptors to engage the inflammatory cascade (Nakashima et al., 1999; Sun et al., 2003). These complexes are recognized by cell surface receptors, or if small enough, phagocytosed altogether (Nakashima et al., 1999; Purdue et al., 2007). Although, numerous biological pathways in macrophages, fibroblasts and other cells are involved in these events, the key molecules involved in particle-associated inflammation include the adapter protein Myeloid Differentiation primary response gene 88 (MyD88), and the transcription factor nuclear factor kappa-light-chain-enhancer of activated B cells (NFκB) (Nakashima et al., 1999; Clohisy et al., 2004; Ren et al., 2004; Baumann et al., 2005; Pearl et al., 2011). Activation of MyD88 and NFκB lead to the transcription of numerous pro-inflammatory substances and upregulation of the innate and (to a lesser degree with respect to wear particle disease) the adaptive immune systems (Pearl et al., 2011; Landgraeber et al., 2014; Nich et al., 2016). In bone and the surrounding tissues, this results in an influx of primarily monocyte/macrophages, but also mast cells, polymorphonuclear leukocytes, T lymphocytes, osteoclasts and other cells are present (Hallab and Jacobs, 2017). The resulting pro-inflammatory environment leads to increased bone destruction by cells of the monocyte/macrophage/osteoclast lineage and suppressed bone formation by cells of the mesenchymal stem cell (MSC)/osteoblast lineage (Kadoya et al., 1996; Vermes et al., 2000; Jacobs et al., 2001). With regards to osteoclastogenesis, the Receptor Activator of Nuclear Factor-kappa B Ligand (RANKL)-RANK- osteoprotegerin (OPG) axis becomes dysregulated, leading to increased osteoclast formation and activation (Haynes et al., 2001). Furthermore, soluble and particulate cobalt-chrome molybdenum alloy (and other particle types) are capable of activating the intracellular inflammasome pathway which increases the secretion of IL-1 and other pro-inflammatory cytokines (Caicedo et al., 2008). As more wear particles are continuously produced with use of the artificial implant, the acute inflammatory reaction becomes chronic, with progressive synovitis and bone destruction. In addition, the presence of endotoxin on the particles and other bacterial byproducts can sustain and exacerbate the inflammatory reaction (Bi et al., 2001).

## *In vitro* and *in vivo* Models of Particle-Induced Inflammation Suggest Potential Avenues for Treatment

In general, our tact has been to develop *in vitro* models for proof-of-principle testing of new concepts and biologics, and then expand and validate these hypotheses using *in vivo* models that simulate the biological events of wear particle disease as closely as possible. One appreciates the associated temporal compression of such models compared to a disease in humans that usually takes many years to develop. Moreover, in investigating the resultant inflammatory bone loss associated with wear particles, one also recognizes the suppressive effects of particles on MSC-osteoblast lineage cells (Wang et al., 2002; Chiu et al., 2006, 2009; Goodman et al., 2006; Ramachandran et al., 2006; Atkins et al., 2009; Pajarinen et al., 2017a). This realization has led to novel methods not only to mitigate bone destruction, but to enhance bone formation, subjects very relevant to the broader topics of tissue engineering and repair of bone. It is also recognized that the pro-inflammatory effects associated with wear particles are not the only factors leading to dysregulated bone biology around joint replacements; other factors include the presence of bacterial ligands, mechanical forces, fluid pressure, and immune reactions especially to metal byproducts etc. (Aspenberg and Herbertsson, 1996; Aspenberg and Van der Vis, 1998; Bi et al., 2002; Cho et al., 2002; Choi et al., 2005; Caicedo et al., 2008; Greenfield and Bechtold, 2008).

Numerous studies have established that wear particles both upregulate the inflammatory cascade and suppress the pathways that facilitate bone formation (Jacobs et al., 2001; Goodman, 2007; Purdue et al., 2007; Goodman and Ma, 2010). *In vivo* models of particle induced osteolysis have the difficulty of simulating a complex series of biological events in a short period of time, in a cost-effective and practical manner. Nonetheless, both small and large animal models have demonstrated some of the important pathogenetic mechanisms leading to particle-associated osteolysis (Lind et al., 1998; Cordova et al., 2014; Moran et al., 2017).

Originally, our laboratory used simpler models encompassing a single bolus of different particles alone, or with more basic implants resurfacing only one side of a joint, or in bone harvest chambers in rabbits; we also implanted particles around a solid intramedullary rod in mice (Goodman et al., 1993; Goodman, 1994; Sacomen et al., 1998; Epstein et al., 2005b; Zilber et al., 2008). While these models provided important information regarding the acute inflammatory reaction to particles (which perhaps was more relevant to the bedding in phase of wear and osseointegration of implants), there were several deficiencies. First, particles are continuously produced from bearing surfaces in human joint replacements and a single bolus of particles does not simulate this scenario. Second, the cellular processes reflective of more chronic particle exposure and the longer-term attempts at re-establishment of tissue homeostasis could not be investigated. Third, some of the models, such as the calvarial model (using a flat bone) were anatomically and physiologically dissimilar from the clinical situation in which human implants are placed in long bones that have a different anatomical and biomechanical structure, and blood supply. Furthermore, the calvarial model does not usually use an implant to simulate a prosthesis. Fourth, the rabbit models were expensive and proved difficult to use with cutting-edge technologies such as genetic manipulation of cells, advanced imaging techniques etc. Nevertheless, single bolus models are still relevant, as they have demonstrated that wear particles of different materials stimulated a macrophage dominated foreign body inflammatory reaction that increased bone destruction and diminished bone formation. The key pro-inflammatory cytokines (TNFα, IL-1β, IL-6, and others) and chemokines (MCP-1, etc.) associated with this reaction were identified (Trindade et al., 1999; Epstein et al., 2005a,b). Using these models, we investigated potential treatments for osteolysis, such as the effects of oral non-steroidal anti-inflammatory medications, an oral p38 mitogen-activated protein kinase (MAPK) inhibitor, and locally placed growth factor (e.g., Transforming Growth Factor beta) (Goodman et al., 1999; Kumagai et al., 2008). However, these substances also adversely affected bone formation, and the timing of delivery and optimal dosage were difficult to establish *in vivo*.

**Figure 2:**
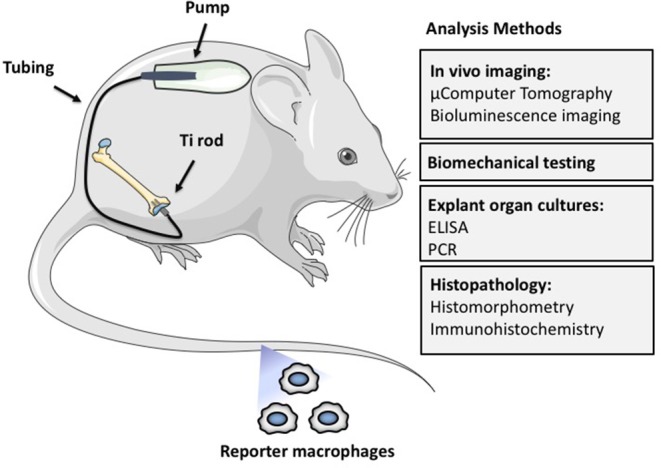
The murine femoral continuous intramedullary particle infusion model. First, the osmotic pump is loaded with biomaterial wear debris and then implanted in the subcutaneous tissue at the dorsum of the mouse. The pump is then connected via subcutaneous tubing to a hollow titanium rod that has been press fit into the intramedullary canal of the distal femur. This arrangement facilitates continuous delivery of biomaterial wear debris to the intramedullary space for 28 days, resulting in continued low grade inflammation and bone loss. The particle delivery can be further extended by changing the pump in a minor surgery. The resulting bone loss at the distal femur can be quantified by imaging techniques such as μCT, biomechanical testing of the peri-implant bone, and histomorphometry. The chronic inflammatory reaction can be quantified by analysis of femoral explant cultures and various histopathological techniques including identification of specific cell populations and their activation states by immunohistochemistry. Finally, systemic homing of macrophages and other cells to the area of inflammation can be quantified by utilizing luciferase labeled reporter macrophages that are injected into the circulation via the tail vein. Adding biologics to the pump with the particles allows the study of potential therapeutic effects of different locally infused treatments.

As a result, more representative models of continuous particle delivery over a more extended time period in small rodents were developed by our laboratory. These models were less costly, simulated the clinical scenario more closely, and could take advantage of newer genetic and imaging technologies. Thus, we developed the murine femoral continuous intramedullary particle infusion model, in which a diffusion pump implanted in the subcutaneous paraspinal region was connected via tubing to a hollow titanium rod placed in the intramedullary canal of the distal third of the femur (Figure 2). Particles and potential therapeutic agents could be loaded into the pump and continuously delivered into bone via the hollow rod over ~28 days. The model was validated first *ex vivo*, prior to its use in live animals (Ortiz et al., 2008a,b). Using histomorphometry, immunohistochemistry, and microCT analysis, we then reported that continuous infusion of clinically relevant polyethylene particles produced a chronic inflammatory macrophage dominated reaction and decreased local bone volume, compared to infusion of the carrier alone (Patterson et al., 2008). Recently we demonstrated that extending the particle delivery time up to 56 days leads to further evolution of chronic inflammation, with continued macrophage activation and bone loss, similar to the progressive clinical scenario (Pajarinen et al., 2017b). We also extended this model to study systemic macrophage trafficking by injecting genetically altered reporter macrophages into the tail vein immediately after surgery, and repeatedly tracked the migration of these cells throughout the body non-destructively via bioluminescence (Ren et al., 2010). To follow systemic trafficking of reporter MSCs, we needed to develop another technique, using left ventricular cardiac cell injection in the beating heart, because the much larger MSCs delivered through the tail vein would sequester in the pulmonary microvasculature, rather than pass through the lungs into the arterial system (Fritton et al., 2012). From these experiments we learned the following: (a) infusion of the chemokine MCP-1 or polyethylene particles via the osmotic pump induces systemic recruitment of reporter macrophages to the local area which results in osteolysis. This macrophage reporter cell trafficking and bone loss could be mitigated by interrupting the MCP-1-CCR2 chemokine-receptor axis using an MCP-1 receptor antagonist or reporter cells from knockout mice that do not possess the CCR2 receptor (CCR2^−^ cells) (Gibon et al., 2012a); (b) luciferase expressing reporter MC3T3 pre-osteoblasts injected into the left ventricle migrated systemically to the area of particle infusion in the distal femur and were associated with increased bone mineral density and markers of bone turnover locally. These effects could be mitigated by injection of an inhibitor of the C-C chemokine receptor CCR1, which interferes with both leukocyte and MSC chemotaxis (Fritton et al., 2012; Gibon et al., 2012b). The above interventions revealed the local and systemic pathways associated with particle-associated inflammation and suggested potential mechanistic interventions for treatment.

**Figure 3:**
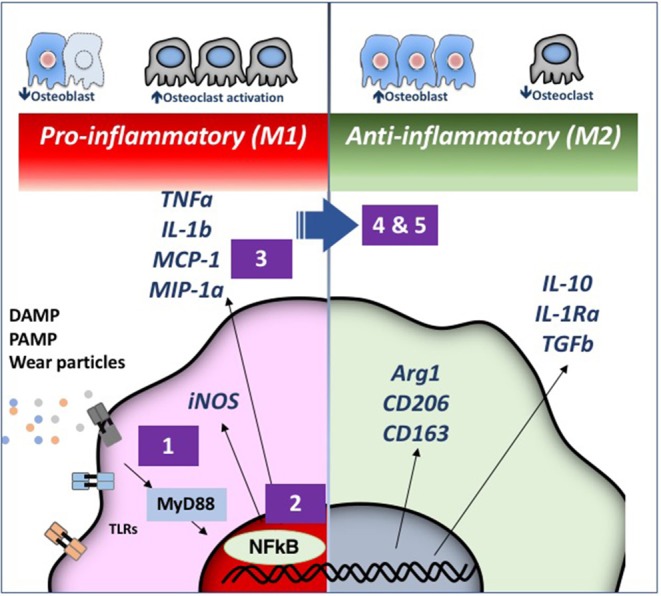
Strategies for immunomodulation to mitigate periprosthetic osteolysis induced by wear particles. Wear particles and adherent pathogen-associated molecular patterns (PAMPs) and byproducts of cell death and tissue injury (damage-associated molecular patterns or DAMPS) can be recognized by Toll-like Receptors (TLRs) and other receptors on macrophages, which then activate downstream pathways including the key transcription factor Nuclear Factor-kappa B (NFκB). The induced pro-inflammatory responses driven by NFκB activation include the expression of inducible nitric oxide synthetase (iNOS) and cytokines/chemokines including Tumor Necrosis Factor alpha (TNFα), Interleukin 1 beta (IL-1b), Macrophage Chemotactic Protein 1 (MCP-1), Macrophage Inhibitory Protein 1 alpha (MIP-1α), and others. These events may lead to periprosthetic osteolysis due to reduced osteoblast and increased osteoclast activity. We have demonstrated that the particle-induced osteolysis can be mitigated by inhibiting (1) the TLR pathway; (2) NFκB activation; or (3) macrophage migration using a mutant MCP-1 called 7ND recombinant protein. Alternatively, pro-inflammatory macrophages (M1, **Left**) can be polarized by (4) Interleukin 4 (IL-4) treatment or (5) genetically modified or preconditioned mesenchymal stem cells (MSCs) (see Figure 4 for details) into an anti-inflammatory, pro-tissue repair macrophage (M2) phenotype (**Right**). M2 macrophages are identified by their expression of Arginase 1 (Arg1) and the surface markers CD206 and CD163. M2 macrophages produce Interleukin 10 (IL-10), IL-1 receptor antagonist (IL-1ra), and Transforming Growth Factor beta (TGFβ). Myeloid Differentiation primary response 88 (MyD88) is a universal adapter protein that is downstream of nearly all TLRs (except TLR3), and leads to activation of NFκB.

More recently, we have engaged 3 strategies to decrease particle associated bone destruction using our murine models (Figure 3). First, we have coated the distal femoral intramedullary rod with a mutant MCP-1 (MCP-1 is also referred to as CCL2) protein called 7ND recombinant protein via a layer-by-layer (LBL) technique to function as a drug eluting device to decrease macrophage trafficking locally (Keeney et al., 2013). Using microCT, immunohistochemical staining, and bioluminescence imaging, local delivery of 7ND protein via the LBL coating decreased systemic reporter macrophage recruitment to the particle infusion area, decreased the number of osteoclasts locally, and mitigated wear particle-induced bone loss in the distal femur (Nabeshima et al., 2017).

Our second strategy was to interfere with the master transcription factor NFκB, which regulates the expression of pro-inflammatory cytokines and chemokines of the innate immune system, and if persistently activated, leads to decreased bone formation and increased bone destruction. We have accomplished this downregulation of NFκB via local infusion of an NFκB decoy oligodeoxynucleotide (ODN), a synthesized duplex DNA that suppresses NFκB activity through competitive binding. We have confirmed the effectiveness of this strategy in *in vitro* studies, and *in vivo*, using the murine calvarial model and the femoral intramedullary particle infusion model (Lin et al., 2014, 2017a; Sato et al., 2015).

Our third strategy is to polarize local macrophages temporally, from an initial pro-inflammatory phenotype (also called M1) to an anti-inflammatory pro-regenerative (M2) phenotype. We accomplished this by exposing the M1 macrophages to interleukin-4, an anti-inflammatory cytokine. *In vitro* studies were first performed in co-culture of undifferentiated macrophages (M0), M1, or M2 together with pre-osteoblasts to determine the optimum time and concentration of cells and IL-4 to optimize bone formation. Polarizing M0 or M1 macrophages to M2 macrophages by the addition of IL-4 optimized matrix mineralization at 3 weeks, and osteocalcin and alkaline phosphatase expression, if the IL-4 was added after ~72 h (Loi et al., 2016b; Córdova et al., 2017). Adding IL-4 earlier or continuously was less optimal. This finding substantiated the belief that a given period of inflammation and osteoprogenitor priming was necessary for optimizing bone formation (Gerstenfeld et al., 2003). After further *in vitro* validation, we subsequently showed that local delivery of IL-4 protein decreased the inflammatory response to particles, and increased net bone formation using the calvarial and the femoral intramedullary particle infusion models (Nich et al., 2013; Pajarinen et al., 2015, 2017b; Sato et al., 2016).

Thus, 3 local potentially translational strategies for modulation of the innate immune system in response to particle challenge were shown to mitigate the adverse inflammatory response and augment bone formation. Although, wear particle disease involves both local, and to some degree, systemic activation of innate immune processes, our group has focused on developing treatment options that are applied locally, directly to the site of the particle induced inflammation; this approach concentrates on altering the biological sequelae of particle disease directly at the source of the problem thereby limiting potential systemic toxicity of the treatments. These potential treatments might have a role in the early stages of osteolysis, when the prosthesis is still salvageable. This biologically based approach supplements ongoing innovations in material science and tribology of joint replacements.

## Modulation of Inflammation: Relevance to Tissue Engineering and Bone Healing

As stated previously, inflammation is the first stage of healing for all tissues. Interestingly, aging is associated with a state of ongoing low grade inflammation (“inflammaging”), and dysregulated macrophage polarization in response to potentially injurious stimuli (Mahbub et al., 2012; Gibon et al., 2016). In other words, with aging, an injury does not always result in a measured coordinated inflammatory reaction with subsequent resolution and repair, but may develop into a chronic inflammatory state with ongoing tissue destruction. Furthermore, aging is associated with a general decrease in the response of both the adaptive and innate immune systems to adverse stimuli (Frasca and Blomberg, 2015). These facts may explain the delayed and/or insufficient healing in the elderly when subjected to traumatic injuries or other adverse stimuli including communicable diseases.

The immune system and the musculoskeletal systems are intimately co-dependent (Loi et al., 2016a). Crosstalk between macrophages and other hematopoietic cells, and MSC lineage cells is important to hematopoiesis, immunomodulation, and the resolution of inflammation, as well as the healing and repair of musculoskeletal tissues (Maggini et al., 2010; Mountziaris et al., 2011; Guihard et al., 2012; Mantovani et al., 2013; Wu et al., 2013; Vi et al., 2015; Loi et al., 2016b). We and others have shown that continuous crosstalk between macrophages and MSC lineage cells are critical to bone healing (Mountziaris et al., 2011; Omar et al., 2011; Vi et al., 2015; Loi et al., 2016b). In addition, with aging, osteogenesis by MSC lineage cells is depressed; these effects have been shown by our group to be associated with ongoing upregulated NFκB activity by aged MSCs (Lin T. H. et al., 2017). Thus, one potential approach to facilitating bone healing in the elderly might be local/regional modulation of NFκB activity in macrophages, directly or indirectly. This approach has been alluded to above.

**Figure 4:**
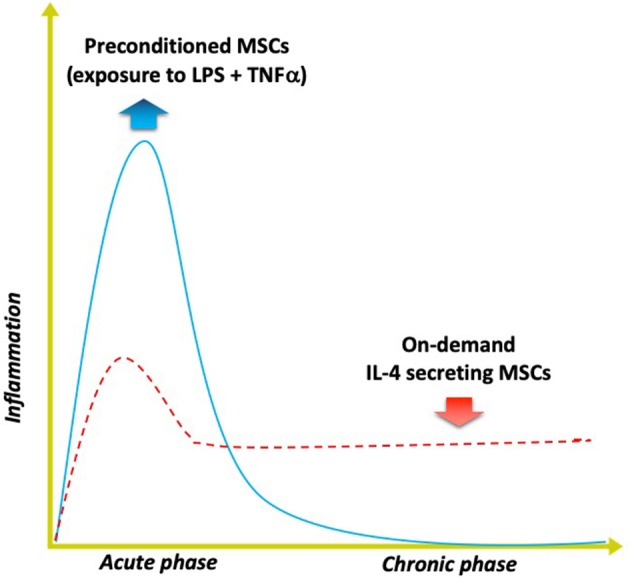
Modulating inflammation with specialized MSCs to enhance bone formation. Optimal bone regeneration is mediated by a transient acute inflammatory reaction (for several days), followed by the resolution of inflammation and the tissue repair phase (blue solid line). Impairment or dysregulation of the acute inflammatory phase may lead to unresolved chronic inflammation and subsequent delayed bone healing (red dashed line). The strategy of using MSCs preconditioned by exposure to both lipopolysaccharide (LPS) plus TNFα *ex vivo* mimics the acute phase response and enhances the MSCs' osteogenic and immunomodulating abilities. Alternatively, genetically modified MSCs that first sense NFkB activation then over-express IL-4 secretion (“on demand”) can respond to unresolved chronic inflammation by modulating the local conditions into the desired anti-inflammatory, tissue repair environment for improved bone healing.

Two additional approaches to immunomodulation by altering MSCs to improve bone healing in inflammatory clinical scenarios have been explored by our group (Figure 4). These approaches are potentially relevant to bone repair in the young and aged alike.

The first approach includes preconditioning the MSCs prior to their use, mimicking the inflammatory environment to which the MSCs are exposed when they first enter the area of tissue damage and regeneration. Other laboratories have demonstrated that preconditioning of MSCs by exposing them to inflammatory cytokines including interferon gamma (INFγ) and TNFα synergistically enhances their immunomodulatory properties by suppressing the activation of T cells (Ren et al., 2008; François et al., 2012). We developed a novel method of preconditioning of MSCs for bone healing applications, using a combination of lipopolysaccharide (LPS- a constituent found in the cell wall of gram negative bacteria) together with TNFα (Lin et al., 2017b). When these preconditioned MSCs (pMSCS) were co-cultured with macrophages, the macrophages polarized from an M1 to an M2 phenotype and were associated with increased osteogenic differentiation of the MSCs, and greater alkaline phosphatase expression and matrix mineralization. Given the fact that inflammation is often part of recalcitrant bone infections, non-union of fractures, periprosthetic osteolysis, osteonecrosis, and other diseases of bone, preconditioning of MSCs may have a direct translational application in the healing of acute and chronic bone defects. Furthermore, the preconditioning protocol developed in our laboratory may prove useful for immunomodulation of other systemic inflammatory disorders such as sepsis, rejection of solid organ transplants etc. in which MSCs are infused.

The second approach encompasses genetic modification of MSCs to over-express the immune-modulating pro-regenerative cytokine IL-4. We have developed 2 constructs to accomplish this goal. In one construct, overexpression of IL-4 by MSCs is continuous; in the other construct, IL-4 is only overexpressed by MSCs when NFκB activity is first sensed as upregulated (Lin et al., 2017c). In the latter construct, when NFκB activity diminishes, the excess production of IL-4 is stopped. Thus, when an inflammatory stimulus is encountered, these genetically modified MSCs (GM-MSCs) can secrete increased amounts of IL-4, subsequently polarizing M1 macrophages (in the vicinity) to an M2 phenotype. Because acute traumatic conditions or adverse stimuli require an initial pro-inflammatory environment to precondition or license the local MSCs for bone healing or other immunomodulatory functions, the IL-4 secreting MSCs would be most useful several days after acute injury, or in chronic inflammatory conditions. The advantage of the NFκB sensing IL-4 overexpressing MSCs is that the delivery of IL-4 could be temporally and spatially tailored to an ever changing inflammatory and immune environment, i.e., be context dependent.

## Discussion

Acute and chronic inflammation are biological processes within the immune system that are integral to the sustenance of life for all organisms. In humans, the innate and adaptive immune systems are highly developed. The former (innate immunity) responds to injury or adverse stimuli in a pre-determined, non-specific manner that is generally dependent on the interaction of cells with chemical motifs that comprise the adverse stimulus. The latter (adaptive immunity) is dependent on the interaction of specific receptors on cells (antigen presenting cells as well as T and B lymphocytes) with a more specific antigenic stimulus. Previously it was thought that only the adaptive immune system had the potential for memory of a previously encountered stimulus challenge; it is now recognized that the innate immune system has a mechanism that “remembers” previous interactions (Italiani and Boraschi, 2017). With subsequent challenges by the same or similar stimuli, monocytes/macrophages can increase (“trained immunity”) or decrease (“tolerance”) the production of cytokines, chemokines, and other substances to effectively deal with a potentially injurious event (Dobrovolskaia and Vogel, 2002). This non-specific innate immune memory can last for months and allows monocytes/macrophages to modulate their functional state according to the persistence of the adverse stimulus. This innate immune memory optimizes survival of the organism by facilitating a relatively speedy and enhanced reaction to potentially harmful stimuli, but also allows a measured defensive response that does not consume the organism (Medzhitov et al., 2012). We are currently exploring these concepts, but much work remains in this paradigm-changing research. For example, it may be possible to create implants that release specific substances based on local contextual cues (e.g., the presence of bacterial ligands or excessive amounts of wear debris); these released substances would then precondition local MSCs or other cells to undertake specific immunomodulatory activities.

How are the above concepts related to wear particle disease? Wear particles are continuously produced by orthopedic implants with repeated usage. In general, debris from commonly used polymers, ceramics and metals in orthopedics provoke an innate immune response; in some cases, protein-metallic byproducts can also act as haptens, thereby stimulating the adaptive immune system as well. Thus, the biological reaction to wear particles from orthopedic implants can function as a paradigm for exploring the mechanisms associated both with acute and chronic inflammation and activation of the immune system using relevant *in vitro* and *in vivo* models. Once these biological processes are elucidated, it may be possible to (a) optimize the composition and design of biomaterials and implants, and (b) modulate tissue-implant responses to facilitate integration of the device or otherwise improve its function *in vivo* in the short and long terms. For joint replacements specifically, these concepts can be translated from bench to beside. For example, implants could be coated with biological substances to facilitate and even expedite initial osseointegration and promote early physiological loading, thus providing pathways for earlier return to function. Methods to mitigate infection, one of the leading causes of implant failure, need to be addressed. This might be accomplished using newer fabrication (for example 3D printing) and coating techniques; alternatively, the periprosthetic environment could be manipulated immunologically to minimize bacterial colonization and expansion. The techniques above to enhance osseointegration and prevent infection could be combined with novel methods to interrogate and sense the periprosthetic environment and then release specific diagnostic and therapeutic agents on demand. These and other interventions would require extensive *in vitro* and *in vivo* testing using relevant animal models. Many of the immune modulating interventions discussed above have only been delivered in the short term. Longer term studies outlining strategies for resolving inflammation at an appropriate time point, without local or systemic adverse effects are needed. Furthermore, novel strategies are needed to address continuous particle production and chronic inflammation over many decades, and potential methods to facilitate particle clearance. Indeed, continuous long-term immunomodulation may have deleterious effects to the host. Thus, solutions will undoubtedly entail better methods of diagnosis of particle-associated inflammation including potential biomarkers that are more sensitive than conventional radiographs, computed tomography, or MRI. In this way, biological interventions could be delivered intermittently at timepoints of higher particle loads and inflammatory responses.

An understanding of the constant interactions among cells of the monocyte-macrophage-osteoclast lineage and the MSC-osteoblast lineage also is critical to tissue engineering of bone. Indeed, the processes of inflammation and bone and soft tissue healing are so intertwined, that impairment of one process impacts the other (Guihard et al., 2012; Mantovani et al., 2013; Loi et al., 2016a,b). Thus, there are significant opportunities for modulating inflammation to obtain a desired outcome for bone healing and regeneration (Mountziaris et al., 2011).

By studying wear particle disease and related pathologies of bone, our group and others have begun to understand the cellular and molecular processes associated with inflammation and activation of the innate immune system and in particular, their role in the formation and destruction of bone. This understanding has led to the design of innovative *in vitro* and *in vivo* models to simulate the activities of the innate immune system and develop potential local treatments to mitigate injurious stimuli and facilitate bone maintenance and repair. As the crosstalk between the innate immune system and MSCs is so critical to bone and soft tissue modeling, investigating ways to optimize their communications has been a continued focus of our current investigations.

On a broader level, innate immune processes and interaction with MSCs are part of a much larger domain. Innate immune cells and MSCs play a major role in the regulation and repair of all cells in the body. Thus, concepts such as modulation of local and systemic cell trafficking, NFκB activity and macrophage polarization provide potential biological strategies for improved clinical outcomes in a variety of diseases that affect virtually every organ system in the body. Thus, from our initial intentions of developing concepts and methods to better understand wear particle disease, our research goals have broadened significantly in order to elucidate and design novel systems for tissue engineering and regenerative medicine. It is hoped that continued research will not only improve the outcome of current and future joint replacements, but provide tangible, evidence-based translational strategies for improving the healing and repair of other organ systems in the body.

## Author Contributions

All authors contributed to the initial concepts, experimental design and methodology, analysis of results and writing of the present manuscript.

### Conflict of Interest

The authors declare that the research was conducted in the absence of any commercial or financial relationships that could be construed as a potential conflict of interest.
